# Identification, classification, and documentation of drug related problems in community pharmacy practice in Europe: a scoping review

**DOI:** 10.1007/s11096-024-01834-7

**Published:** 2025-01-08

**Authors:** Danielle Hochhold, Lotte Stig Nørgaard, Derek Stewart, Anita Elaine Weidmann

**Affiliations:** 1 Department of Clinical Pharmacy, Leopold-Franzens Universität, Innsbruck, Austria; 2 Department of Pharmacy, University of Copenhagen, Copenhagen, Denmark; 3 College of Pharmacy, QU Health, Qatar University, Doha, Qatar

**Keywords:** Classification, Community pharmacy services, Delivery of health care, Documentation, Drug related problems, Identification, Practice patterns

## Abstract

**Background:**

Drug-related problems (DRPs) are significant issues in healthcare contributing to adverse health outcomes and increased healthcare costs. While community pharmacists play a pivotal role in identifying, classifying, and documenting DRPs, there is a need to map approaches undertaken.

**Aim:**

The aim of this scoping review was to examine the approaches to identifying, classifying, and documenting DRPs in community pharmacies in Europe, and their associated barriers and facilitators.

**Method:**

The scoping review was conducted according to the Joanna Briggs Institute guidelines and reported according to the PRISMA-ScR guidelines. The search was conducted across 11 databases from inception until March 2023. Studies of all designs reporting DRPs in European community pharmacies were included. Titles, abstracts, and full texts were screened independently by two researchers, followed by data extraction and synthesis.

**Results:**

The search yielded 67 relevant studies. Forty-eight studies described approaches to DRP identification as predominantly relying on professional knowledge and computer software. The classification methods, described in 41 studies, varied with reports of predefined systems and computer-generated classifications. Documentation approaches were described in 53 studies and were primarily computer-based. Facilitators included electronic support systems, pharmacist experience, while barriers encompassed the complexity of classification as well as variations in training, IT solutions, operational structures, and implementation.

**Conclusion:**

There is a lack of a standardized approach to identifying, classifying, and documenting DRPs in European community pharmacies. A framework for pharmacist education and a time-saving approach to documenting DRPs consistently could serve to overcome the barriers to their identification and documentation. Furthermore, the implementation of a standardised approach to classifying DRPs could facilitate comparison of the management of DRPs across Europe.

**Supplementary Information:**

The online version contains supplementary material available at 10.1007/s11096-024-01834-7.

## Impact statements

- This scoping review illustrates the lack of standardization in identifying, classifying, and documenting drug-related problems (DRPs) across European community pharmacies. It highlights the need for a comprehensive understanding of regional differences to standardise approaches.
- The barriers identified in this research highlight areas of further research focusing on aspects of training, IT solutions, and operational structures.
- The variation in approaches to DRP identification, classification, and documentation underscores the need for standardized education and training programs for pharmacy professionals.

## Introduction

According to the Pharmaceutical Care Network Europe (PCNE) Classification V 9.1, a drug-related problem (DRP) is "*an event or circumstance related to drug therapy that actually or potentially affects desired health outcomes*” [1]. DRPs contribute to patient harm, increased hospital admissions and increased healthcare costs, with studies indicating that a significant portion of hospital admissions, particularly among older patients, can be attributed to DRPs, many of which are preventable [2–6]. A meta-analysis in 2020 found that 3% of patients across all healthcare settings experience preventable medication harm, and another study from 2021 estimated the cost of preventable adverse drug events in England at £98,462,582 per year. [7, 8]. In recent years, numerous studies have focused on the prevalence of DRPs and the clinical and economic outcomes of DRPs in hospital pharmaceutical care, but research for community pharmacies is still scarce [3, 9–12]. Addressing DRPs through pharmaceutical care in community pharmacies places significant demands on the organizational structure and procedural aspects of service delivery within community pharmacies [1, 13–16]. The delivery of pharmaceutical care by community pharmacists varies in scope and advancement throughout Europe [13]. Research demonstrates disparities in pharmaceutical care provision among community pharmacies across European nations, as evidenced by studies highlighting the continued limited availability of pharmaceutical care services in this setting [17–20].

The process of managing DRPs in community pharmacy requires their accurate identification, categorization, and documentation [21, 22]. The identification of DRPs is a multifaceted process involving the collaboration of healthcare professionals. The challenges for pharmacists include identifying DRPs and vulnerable patient groups as well as medications at-risk associated with DRPs [3, 23, 24]. Once identified these DRPs should be allocated to distinct categories of problems, causes and interventions using published classification systems [25–27]. This categorization provides a structured approach to the analysis of DRPs thereby facilitating greater understanding of underlying causes and future prevention [27]. Published validated classification systems used in community pharmacies across Europe include, for example, the Pharmaceutical Care Network Europe (PCNE) Classification, the Westerlund classification, the PI-Doc, the GSASA and the ClinPhADoc [25–29]. Well-structured classification systems have clear definitions for each category, are easy to use, have a clear hierarchical structure and a comprehensive description of the causes of DRPs [30]. Their use has been shown to improve effectiveness (how well it works) and efficiency (how well it is performed) of pharmaceutical care in addition to inter-professional communication [31–33]. The validation of the classification instruments is important to ensure that the desired information is accurately and comprehensively collected and that the instrument is easily understood by pharmacists, researchers, and the wider healthcare team [34, 35]. The published classification systems listed differ in their definition and causes of DRPs, and in their applicability across different healthcare settings [36]. Although many studies highlight that community pharmacists recognize and respond to DRPs, little is published on how community pharmacists identify, classify and document these in practice and the associated facilitators or barriers [18, 27, 37, 38]. The documentation of DRPs is essential for reporting adverse drug reactions, and other drug-related issues, thereby contributing to the safe and effective use of medicines as well as the provision of pharmaceutical care services in community pharmacies.

### Aim

The aim of this scoping review was to examine the approaches of Identification, classification, and documentation of drug related problems in community pharmacy practice in Europe, and their associated barriers and facilitators.

## Method

The scoping review was carried out in following the Joanna Briggs Institute (JBI) guidance for scoping reviews and reported according to the PRISMA ScR reporting guidelines [39, 40]. The scoping review protocol is made available in supplementary material 1.

### Inclusion criteria

Included in the review were studies conducted in community pharmacies in Europe that focused on the identification, classification, or documentation of DRPs. Studies focused only on one or two aspects were also included. Excluded were studies conducted in other healthcare settings or studies focused on medication reviews. Given the context of community pharmacy practice, studies that focused solely on theoretical approaches to the management of DRPs were also excluded.

All study designs, text and opinion papers as well as grey literature such as case reports, editorials, discussion papers, conference proceedings published in peer-reviewed papers, dissertations, and theses were included. Study protocols were excluded. The reference lists of full-text studies were hand-searched to ensure capture of all relevant studies. The search included studies published in English language from database inception to March 2023.

### Search strategy

The search was conducted across 11 different databases (Pubmed, Web of Science, Cochrane library, Social Science Research Network (SSRN), PsycInfo, Open Dissertation, Livivo, OpenAire, ProQuest, Cinahl, IPA) and focused on DRPs (“drug therapy problems”, “medication related problems”, “therapy related problems”, “medication management problem”, “intervention”, “drug related issue”, “drug related finding”, “pharmaceutical care”, “pharmaceutical service”) in community pharmacies across Europe. The final search string was refined with the help from a research librarian at the University of Innsbruck and is provided in supplementary material 2.

All studies were exported to EndNote Web, duplicates removed and all remaining studies exported to Rayyan QCRI® [41]. Screening followed a sequential assessment of titles, followed by abstracts and full papers, completed by two independent researchers (DH and AEW/DS/LSN). Discrepancies were discussed or resolved with the input from a third researcher.

### Data extraction

Data were extracted and summarized using a data extraction form developed by the research team according to the aim of the scoping review and based on the PRISMA-ScR guidance and is provided in the supplementary material 3 [40, 42]. The draft data extraction form was piloted by one researcher (DH) and extraction discussed with a second researcher (AW) at title screening stage. Data extraction was undertaken by one researcher (DH) and independently verified by another researcher (AEW). Disagreements were resolved by discussion with a third researcher (DS/LSN). Extracted data were the study title, date of publication, country, design, population, approaches to identification, classification and documentation of DRPs, reported facilitators and barriers, and key findings.

### Data synthesis

The extracted information was mapped in tabular form and reported descriptively.

## Results

### Study selection

**Fig. 1:**
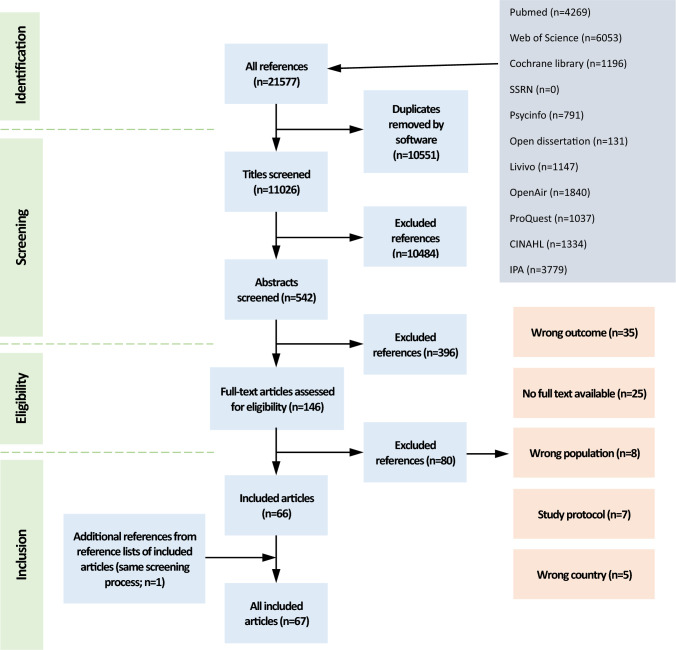
PRISMA scoping review study selection flow chart summarising the process of identification and screening of literature reports for inclusion

The search yielded 11,026 unique records (Fig. 1), which were reduced to 66 studies following screening of titles, abstracts and full text. One study was added from screening reference lists resulting in 67 studies included in the review.

### Study characteristics

Studies originated from the Netherlands (n = 17), United Kingdom (n = 10), Switzerland (n = 7), Spain (n = 6), Germany (n = 5), Denmark, France, Sweden (n = 4 each), Finland (n = 2), and one each from Belgium, Hungary, Malta, Portugal and Turkey. Three studies reported data from a number of different countries (Table 1). Thirty studies did not state the study design. Study designs (according to authors) were observational (n = 6), surveys (n = 6), randomised controlled trials (n = 5), cross-sectional studies (n = 3), retrospective analysis (n = 3), prospective studies (n = 2), quantitative analysis (n = 2), and one each of literature review, pilot study, case control study, follow-up study, service evaluation, 3-phase study and post-hoc analysis.

**Table 1:** Study characteristics as stated by the authors

Study authors; (year); country	Aims	Study design	Participants (No.); Description of participants	Key findings related to scoping review
Stevens RG, Balon D; (1997) [50]; United Kingdom	Detect and record drug-drug interactions	Not stated	All prescriptions (n = 57,853)	136 warnings for 53 patients
Harris et al.; (1998) [64]; United Kingdom	Describe and quantify assessment and referral activity	Not stated	Pharmacists (n = 14); 858 assessments	57% (n = 490) relating to physical symptoms27% (n = 232) prescription medication
Chamba et al.; (1999) [76]; France	Describe the pharmacist’s role to improve pharmacotherapy	Survey	Pharmacists (n = 37); 737 reported acts to improve or correct dispensations	lack of precision of prescription (275), abnormality of dosing (166), interactions between drugs or contraindications (172)
Westerlund et al.; (1999) [14]; Sweden	Document types and number of drug-related problems	Not stated	Patients (39,143)	DRPs in 2.5% (n = 975) of the patients.2/3 were detected by the pharmacy staff
Bernsten et al.; (2001) [77]; 7 European countries	Investigate the impact of a pharmaceutical care programme	Randomised, controlled trial	Patients (1290 intervention, 1164 control group)	Structured service well accepted
Buurma et al.; (2001) [51]; Netherlands	Investigate the frequency, nature and determinants of prescription modifications	Prospective case–control study	Modified prescriptions (2014)	Modifications found three times more often in handwritten prescriptions
Foppe van Mil et al.; (2001) [34]; Netherlands	Quantify the CGAs and the pharmaceutical services	Not stated	Pharmacies (17)	24 ± 8 care activity codes (CACs) per 100 prescriptions
Westerlund et al.; (2001) [28]; Sweden	Document the number and types of DRPs identified (OTC products)	Not stated	All customers buying OTC medication (1418)	The most prevalent DRPs were: uncertainty about the indication for the drug (33.5%, n = 477) and therapy failure (19.5%, n = 278)
Layton et al.; (2002) [60]; United Kingdom	Monitor the safety of an OTC medication	Not stated	Customers purchasing Ibuprofen (1021)	Use of concomitant medicationHigher prevalence of GIT symptoms
Barris et al.; (2003) [78]; Spain	Analyse DRPs detected in community pharmacy	Not stated	Pharmacist (1)	44 drug related problems were detected
Leemans et al.; (2003) [79]; Belgium	Investigate the frequency of interventions for prescription medicine	Not stated	Pharmacists (n = 130)	Clinical interventions less frequently than technical ones
Rossing et al.; (2003) [15]; Denmark	Determine the frequency of medicine-related problem identification	Cross-sectional questionnaire-based survey	Community pharmacists (218)	Medicine-related problems detected in 3 patients per pharmacy, minimal documentation
Sturgess et al.; (2003) [16]; Northern Ireland	Evaluated impact of a pharmaceutical care programme	Randomised, controlled trial	Patients (191: 110 intervention and 81 control)	56,4% of problems associated with compliance (n = 115)
Buurma et al.; (2004) [65]; Netherlands	The assessment of clinical value of prescription-error modifications	Not stated	Prescriptions of community pharmacy (144); modified prescriptions	One in 200 prescriptions (0.49%) was positively modified
Hugtenburg et al.; (2004) [53]; Netherlands	Assess the effect of a short inquiry on the detection of drug-related problems	Not stated	Patients (700)	In 22% (n = 156), drug- related problems mainly concerned side effects (49%; n = 76)
Chen et al.; (2005) [80]; England	Describe prescribing problems	Not stated	Pharmacies (9); prescribing problems (201)	In 2/3 of the problems was incomplete or incorrect information
Buurma et al.; (2006) [81]; Netherlands	Measure drug-drug alerts	Not stated	Community pharmacies (63); drug-drug interaction alerts	First alerts lead to more external action than recurring, different prescriber led to more external action, younger age lead to more external action
De Oliveira Martins et al.; (2006) [82]; Portugal	Inappropriate drug use by Portuguese elderly outpatients	Observational cross-sectional survey	Patients (213); 65 years and older, prescription with 2 or more drugs	Higher rates with new Beers list, the prevalence higher in patients who are taking a higher amount of drugs
Vinks et al.; (2006) [23]; Netherlands	Identify potential DRPs from prescription records of the elderly	Not stated	Patients (196); aged 65 and over using six or more drugs	A total of 763 potential DRPs were observed in the 196 patients
Becker et al.; (2007) [43]; Netherlands	Assess characteristics associated with the dispensing of interacting drug combinations	Not stated	Community pharmacies (286); interacting drug combinations	Different medication surveillance systems differed in the dispensing interacting drug combination
Griese et al. (2007) [21]; Germany	Identify the spectrum of DRPs	Survey	Community pharmacies (1146); documented DRP	Pharmacists documented 10,427 DRPs362 cases could not be classified within this final system
Indermitte et al.; (2007) [44]; Switzerland	Assess the prevalence of potential drug interaction	Not stated	Patient (102) buying out of a defined list of medication	14,4% (n = 64) requested potentially interacting OTC drugs In 15,9% (n = 69) potential drug interactions between OTC drugs and POMs were found
Knudsen et al.; (2007) [67]; Denmark	Measure frequency and types of error	Not stated	Prescriptions (976)	976 cases of prescription corrections, 203 cases of dispensing errors and 198 cases of adverse drug events
Lynskey et al.; (2007) [83]; United Kingdom	Establish the number of medication errors	Not stated	Pharmacies (15); self-reporting form (145)	113 near misses and 32 medication errors were reported
Kraehenbuehl et al.; (2008) [54]; Switzerland	Report and assess routine community pharmacists’ interventions process	Not stated	Patients (38,663); patients with prescriptions	Pharmacists’ interventions mainly concern the resolution or prevention of a dosage or regimen problems, drug–drug interactions, and adherence problems
Krska J, Avery AJ; (2008) [55]; United Kingdom	Describe issues noted and recommendations made by community pharmacists	Randomized controlled trial	Intervention patients (738)	A total of 2228 issues and 2337 recommendations were noted
Montgomery et al.; (2008) [61]; Sweden	Identify characteristics of patients who registered for the pharmaceutical care service	Retrospective assessment of data	PMR data (3298)	25.8% (n = 851) had DRP
Vinks et al.; (2009) [56]; Netherlands	Investigate whether a pharmacist-led intervention scheme reduces the number of potential DRP	Controlled follow-up study	Patients (174, 87 intervention, 87 control);	After a 4-month period, a significant reduction in the mean number of DRPs per patient was observed
Eichenberger et al.; (2010) [45]; Switzerland	Explore the occurrence, nature and pharmacist’s management of drug-related problems	Prospective observational study	Patients (616)	6.1% (n = 141) clinical and 12.0% (n = 278) technical DRPs
Lewinski et al.; (2010) [46]; Germany	An analysis on DRPs, and relevant risk factors	Cross sectional study	Patients (3040)	DRPs were detected in 21,0% of patients (n = 638)
Eickhoff et al.; (2011) [84]; Germany	Quantify and specify DRPs in OTC drug use	Not stated	Customers (11,069)	Pharmacists documented one or more DRPs in 17.6% (n = 2206)
Sanchez and Campos; (2011) [85]; Spain	Detect prescribing-related problems	Descriptive study	Prescriptions (23,995)	355 prescribing errors were detected
Vella and Azzopardi; (2011) [86]; Malta	Performing a detailed examination of the patient’s medication regimen	Not stated	Patients (80)	86% of patients (n = 69) were receiving appropriate treatment for their condition
Rossing et al.;(2012) [68]; Denmark	Provide description of drug-related problems in self-medication	Descriptive study	customers (3868)	In 20% of OTC request DRP were identified, most could be solved
Correr et al.; (2013) [87]	A Tool to Characterize the Components of Pharmacist Interventions	3-phase study	49 reviews	Developed a tool with 12 domains and 54 items
Franklin et al.; (2013) [88]; United Kingdom	Document the numbers and types of interventions	Not stated	Prescriptions in community pharmacies (68)	33 problems were clinical and 6 were organisational or logistical
Griese et al.; (2013) [29]; Germany	Quantify DRPs in prescribed drugs	Not stated	Community pharmacies (130);patients (14,231)	DRPs in 18.0% (n = 2556) of all patients and in 11.2% (n = 2732) of all prescribed drugs
Sanchez; (2013) [89]; Spain	Investigates the nature, frequency, and potential causes of medication errors	Not stated	Individual prescriptions (42,000)	2,117 medication errors; there were 1,127 prescribing errors, 216 dispensing errors, and 774 near-misses
Ahmad et al.; (2014) [24]; Netherlands	Analyse solved and unsolved DRPs detected in community pharmacy patients	Observational study	Patients (340)	992 potential DRP were observed in the 340 patients
Basger et al.;(2014) [36]	Which DRP classification systems have been chosen	Literature review	All (exclusion: ADR, ME, selected types or causes of DRP)	requirements included: an open, hierarchical structure, clear definitions of the term “DRP” and of DRP categories leading to only one choice of coding
Maes et al.; (2015) [35]; Switzerland	Adapt the existing GSASA system to suit the community pharmacy setting	Not stated	All students (n = 77) asked to collect 10 prescriptions	The classification system includes 5 main categories and 52 subcategories
Mast et al.; (2015) [90]; Netherlands	Develop a tool to facilitate and support the periodic review of older patients’ medication	Not stated	Patients older than 65, with chronic conditions	The medication review tool consists of a comprehensive checklist of 124 drug-related problems
Twigg et al.; (2015) [57]; United Kingdom	Describe the effect of a holistic community pharmacy-based service	Service Evaluation	Patients (620); over 65 years old and taking FOMM	142 recommendations to prescribers in 110 patients
Verdoorn et al.; (2015) [62]; Netherlands	Determine the number and types of STOPP/START criteria present in identified drug-related problems	Not stated	Patients (533);	The total number of potential DRPs identified by community pharmacists was 1656 in 457 patients
Chau et al.; (2016) [72]; Netherlands	Investigate the number of DRPs in the elderly with polypharmacy	Cross-sectional	Patients (3807); 65 or older with polypharmacy	A median of two DRPs was identified per patient
Heringa et al.; (2016) [47]; Netherlands	Associations between the drug-drug interaction alerts and drug-disease interaction alerts	Retrospective analysis	Pharmacies (123); computer-generated drug-drug interaction alerts	0.6 million drug interaction alerts, corresponding to an average of 185 drug interaction alerts per pharmacy per day
Messerli et al.; (2016) [63]; Switzerland	Investigating the impact of the PMC on patients on polypharmacy	Randomised controlled trial	Potential PMC candidates (450)	Pharmacists reported 258 drug-related problems (1.18 per patient)
Bourcier et al.; (2017) [58]; France	Assess the quality of prescriptions	Prospective observational study	Patients (1206) older than 75	Among the 1206 prescriptions analysed, 67.49% (n = 814) contained a PIM
Maes et al.; (2017) [27]; Switzerland	Validate the PharmDISC system in the daily practice environment	Prospective observational study	Prescriptions requiring a PI (535)	Of 519 PIs,82,9% (n = 430) were completely classified
Schoenmakers et al.; (2017)[66]; Netherlands	Describe the numbers and types of symptoms assessed during a CMR	Cross-sectional observational study	Patients (118)	1102 symptoms in 22 predefined symptom categories were reported
Seidling et al.; (2017) [69]; Germany	Analyze the impact of medication reviews	Post-hoc analysis	Older patients (912)	In 869 cases the pharmacist documented at least one information need or DRP
Rhalimi et al.; (2018) [70]; France	Describe the number and type of DRPs identified by community pharmacists in elderly patients	Prospective multicentre study	Patients (892)	334 DRPs were identified and were associated with 259 PIs
Verdoorn et al.; (2018) [48]; Netherlands	Investigate the effect of adding a clinical decision support system to medication review software	Retrospective database study including a pre- to post-design	Patients (3100)	9151 DRPs were identified in 3100 patients pre- CDSS and 15 268 DRPs were identified in 4303 patients post- CDSS
Vo et al.; (2018) [91]; France	Develop and validate a tool for recording and classifying PIs	Not stated	Pharmaceutical interventions (60)	The tool developed for recording and classifying PIs has 19 items
Hamada et al.; (2019) [38]; Switzerland	To update the ClinPhADoc tool for the documentation of clinical activities in the community pharmacy	Not stated	Clinical activities (136)	Pharmacists were able to document 131 clinical activities
Verdoorn et al.; (2019) [92]; Netherlands	To determine the impact ofa patient-centred approach in CMR	Randomised controlled trial	Patients (629	The number of health problems with impact on daily life decreased by 12%
Danish et al.; (2020) [49]; Sweden	Investigate whether the use of the pharmacy based EES (Electronic ExpertSupport) would identify andresolve more actual patient DRPs	Prospective, open, controlled, four period comparative study	Patients (200)	In the EES-support group drug-drug interactions were the most common DRP type (33%, n = 17). In the control group it was drug duplications (38%, n = 15)
El-Souri et al.; (2020) [93]; Denmark	Map the pharmacy technicians’ counselling activities	Descriptive study	Customers (17,692)	Identified DRPs for 15.8% of all registered customers
Varas-Doval et al.; (2020) [94]; Spain	Evaluate the impact of an implementation programme for MRF in community pharmacies	Not stated	Patients (608)	55% of pharmacies reached the implementation phase
Varas-Doval. et al. (2020) [95]; Spain	Describe the effectiveness of MRF provision for aged polypharmacy patients	Cluster randomised controlled trial	Patients (1403)	Most prevalent DRPs identified were, undertreated condition (n = 559, 35.81%), lack of treatment adherence (n = 261, 16.7%) and risk of adverse effects (n = 207, 13.26%)
Ylä-Rautio et al.; (2020) [22]; Finnland	Determine the number and nature of OTC-related DRPs	Observational study	Customers (55,296)	DRPs were documented in 0.6% (n = 339) of all OTC customers
Kallio et al.; (2020) [19]; Finland	Investigate community pharmacists’ contributions to medication risk management	Survey	Pharmacies (169)	Pharmacists were oriented to solve poor adherence and technical problems in prescriptions
McCahon et al.; (2021) [96]; United Kingdom	To develop an evidence- based, structured model of medication review for use in clinical practice	Not stated	Articles (32)	Final model considered to benefit from holistic, patient-centred approach
Soler et al.; (2021) [97]; Spain	Evaluate the implementation of the MUR service	Cross-sectional multicentre study	Patients (495)	The pharmacists provided tailored information for 2073 medicines (73.8%) and 1316 suggestions for improving use (46.8%)
Szilvay et al.; (2021) [71]; Hungary	Examine the interaction risks of patients with polypharmacy	A multicenter descriptive study	Patients (755)	984 DRPs (1.3 DRPs per patient) were registered
Van Loon et al.; (2021) [52]; Netherlands	Investigate the nature and frequency of prescription modifications	Cross sectional study	Prescription modifications (2385)	A modification was performed in 5.5% (n = 5385) of all prescriptions
Dal et al.; (2022) [59]; Turkiye	Evaluate clinical pharmacist medication review	Pilot study	Patients (100)	At least one potentially inappropriate medication was detected in 63.0% of them

### Approach to the identification of drug related problems

The approach to the identification of DRPs was described in 48 studies, 18 of which described related pharmacy staff training (Table 2). The most widely used approach relied on the identification of DRPs using the professional knowledge of pharmacy employees (n = 19). The use of computer software to identify DRPs was described in 15 studies, with a plethora of different software systems and approaches reported (n = 15). While a number of studies reported computer generated alerts [21, 34, 43–49] others combined pharmacy interaction software with professional knowledge [50–52] or patient consultation [19, 29, 51]. Eight studies reported the identification of DRPs using prescribing guidelines or medication appropriateness indices [16, 53–59] such as national prescribing guidelines (n = 5), the Beers criteria (n = 2), START/STOPP criteria (n = 1) or the Medication Appropriateness Index (n = 1). Six studies reported other approaches to the identification of DRPs including a patient questionnaire or the identification of specific high-risk medications [19, 28, 29, 51, 53, 60].

**Table 2:** Approaches of identification of DRPs and training provided in community pharmacies in Europe

	Stevens RG, Balon D; (1997) [50]	Westerlund et al.; (1999) [14]	Bernsten et al.; (2001) [77]	Buurma et al.; (2001) [51]	Foppe van Mil et al.; (2001) [34]	Westerlund et al.; (2001) [28]	Layton et al.; (2002) [60]	Barris D, Faus MJ; (2003) [78]	Leemans et al.; (2003) [79]	Sturgess et al.; (2003) [16]	Hugtenburg et al.; (2004) [53]	Buurma et al.; (2006) [81]	De Oliveira Martins et al.; (2006) [82]	Vinks et al.; (2006) [23]	Becker et al.; (2007) [43]	Griese et al. (2007) [21]	Indermitte et al.; (2007) [44]	Knudsen et al.; (2007) [67]	Kraehenbuehl et al.; (2008) [54]	Krska J, Avery AJ; (2008) [55]	Vinks et al.; (2009) [56]	Eichenberger et al.; (2010) [45]	Lewinski et al.; (2010) [46]	Sanchez AM, Campos RM; (2011) [85]
*Training provided*																								
			✔							✔	✔			✔		✔			✔		✔		✔	
Approach to Identification																								
Prescribing Guidelines, predefined criteria													✔	✔					✔	✔	✔			
Computer software	✔			✔	✔							✔			✔	✔	✔					✔	✔	
Professional knowledge of pharmacy workers	✔	✔		✔		✔			✔	✔				✔				✔	✔		✔			✔
Structured medication review			✔					✔												✔				
Other				✔		✔	✔				✔													

	Vella J, Azzopardi L; (2011) [86]	Rossing et al.;(2012) [15]	Griese et al.; (2013) [29]	Ahmad et al.; (2014) [24]	Mast et al.; (2015) [90]	Twigg et al.; (2015) [57]	Verdoorn et al.; (2015) [62]	Chau et al.; (2016) [72]	Heringa et al.; (2016) [47]	Messerli et al.; (2016) [63]	Bourcier et al.; (2017) [58]	Schoenmakers et al.; (2017) [66]	Rhalimi et al.; (2018) [70]	Verdoorn et al.; (2018) [48]	Verdoorn et al.; (2019) [92]	Danish et al.; (2020) [49]	El-Souri et al.; (2020) [93]	Varas-Doval et al.; (2020) [95]	Ylä-Rautio et al.; (2020) [22]	Kallio et al.; (2020) [19]	Soler et al.; (2021) [97]	Szilvay et al.; (2021) [71]	Van Loon et al.; (2021) [52]	Dal et al.; (2022) [59]
Training provided																								
		✔				✔				✔		✔	✔		✔		✔	✔			✔	✔		
Approach to Identification																								
Prescribing Guidelines, predefined criteria						✔					✔													✔
Computer software			✔						✔					✔		✔				✔			✔	
Professional knowledge of pharmacy workers	✔	✔					✔			✔			✔				✔		✔				✔	
Structured medication review				✔	✔			✔				✔	✔		✔			✔			✔	✔		
Other			✔																	✔				

### Approach to the classification of drug related problems

The approach to classification of DRPs was reported in 41studies. Eighteen studies reported the use of a classification form or a predefined list of DRPs. There was heterogeneity in the approaches to classifying DRPs. Of the 15 studies using published classification systems, 7 modified the classification system for their use (Table 3). Overall the PI Doc classification (n = 3) and the Westerlund classification (n = 3) were most commonly used, followed by the PCNE classification (n = 2), DOCUMENT (n = 2) and the GSASA classification (n = 2) [14, 21, 22, 24, 29, 35, 45, 46, 52, 61–63].

**Table 3:** Approaches of classification of DRPs in community pharmacies in Europe

Study	PCNE^a^	DOCUMENT^a^	PROMISE^a^	GSASA^a^	PharmDisc^a^	ClinPhADoc^a^	PI Doc	Westerlund System^a^	Computer based Classifiacation^b^	Classification form (undefinded)^c^	Predefined list of DRPs^d^	Expert panel^e^
Stevens RG, Balon [50]; United Kingdom										✔		
Westerlund et al. [14]; Sweden								✔				
Foppe van Mil et al. [34]; Netherlands									✔			
Barris D, Faus MJ [78]; Spain										✔		
Leemans et al. [79]; Belgium										✔		
Sturgess et al. [16]; Northern Ireland										✔		
Buurma et al. [65]; Netherlands												✔
Hugtenburg et al. [53]; Netherlands										✔		
Chen et al. [80]; United Kingdom											✔	
Vinks et al. [23]; Netherlands										✔		
Buurma et al. [81]; Netherlands												✔
Becker et al. [43]; Netherlands											✔	
Griese et al. [21]; Germany							✔					
Indermitte et al. [44]; Switzerland									✔			
Kraehenbuehl et al. [54]; Switzerland									✔			
Krska, Avery [55]; United Kingdom										✔		
Montgomery et al. [61]; Sweden								✔				
Vinks et al. [56]; Netherlands										✔		
Eichenberger et al. [45]; Switzerland	✔											
Lewinski et al. [46]; Germany							✔					
Sanchez AM, Campos RM [85]; Spain										✔		
Franklin et al. [88]; United Kingdom										✔		
Griese et al. [29]; Germany							✔					
Sanchez AM [89]; Spain											✔	
Ahmad et al. [24]; Netherlands	✔											
Maes et al. [98]; Switzerland				✔								
Twigg et al. [57]; United Kingdom											✔	
Verdoorn et al. [62]; Netherlands		✔										
Heringa et al. [47]; Netherlands									✔			
Messerli et al. [63]; Switzerland				✔								
Maes et al. [27]; Switzerland					✔							
Schoenmakers et al. [66]; Netherlands			✔									
Seidling et al. [69]; Germany											✔	
Rhalimi et al. [70]; France										✔		
Verdoorn et al. [48]; Netherlands									✔			
Hamada et al. [38]; Switzerland						✔						
Danish et al. [49]; Sweden										✔		
Ylä-Rautio et al. [22]; Finnland								✔				
Szilvay et al. [71]; Hungary										✔		
Van Loon et al. [52]; Netherlands		✔							✔			

^a^previously published classification of drug related problems (DRPs), an asterisk indicates that the classification form has been modified for the use in the study

^b^classification was done using a computer system

^c^classification was done using a form but not described in detail in the study

^d^study authors used a predefined list of drug related problems

^e^an expert panel was used to classify DRPs

### Approach to the documentation of drug related problems

The majority of the studies (n = 53) described an approach to documenting DRPs (Table 4). While 24 out of 53 studies did not specify their approach, 6 studies described a paper-based documentation system [14, 38, 53, 64–66] and 23 studies detailed the use of a computer-based approach. In 11 of these computer-based documentation systems, a pharmacy staff member completed an electronic form [15, 19, 22, 28, 43, 57, 66–71] while documentation was automatically performed by software in 4 studies [34, 47, 48, 50]. In 8 studies no further details to the computer-based approach were provided.

**Table 4:** Approaches of documentation of DRPs in community pharmacies in Europe

	Stevens RG, Balon D; [50]	Harris et. al.; [64]	Westerlund et. al. [14]	Bernsten et. al. [77]	Buurma et. al. [51]	Foppe van Mil et. al. [34]	Westerlund et. al. [28]	Layton et al. [60]	Leemans et. al [79]	Rossing et. al. [15]	Sturgess et. al. [16]	Buurma et. al. [65]	Hugtenburg et. al. [53]	Chen et. al. [80]	Buurma et. al. [81]	Vinks et. al.; [23]	Griese et. al. [21]	Indermitte et. al. [44]	Lynskey et. al. [83]	Becker et. al. [43]	Knudsen et. al [67]	Krska J, Avery AJ [55]	Kraehenbuehl et. al. [54]	Montgomery et. al. [61]	Vinks et. al. [56]	Eichenberger et. al. [45]	Lewinski et. al. [46]
Documentation stated but not defined				✓	✓			✓	✓		✓			✓	✓	✓	✓	✓	✓			✓			✓	✓	✓
Paper-based documentation		✓	✓									✓	✓														
Computer based documentation	✓					✓	✓			✓										✓	✓		✓	✓			
User completed electronic form							✓			✓										✓	✓						
Automatically generated by computer	✓					✓																					

	Eickhoff et. al. [84]	Sanchez AM, Campos RM [85]	Vella and Azzopardi [86]	Rossing et. al. [68]	Franklin et. al. [88]	Griese et. al. [29]	Sanchez [89]	Twigg [57]	Chau et. al. [72]	Heringa et. al. [47]	Messerli et. al. [63]	Bourcier et. al. [58]	Maes et. al. [27]	Schoenmakers et. al. [66]	Seidling et. al [69]	Rhalimi et. al. [70]	Verdoorn et. al. [48]	Vo et. al. [91]	Hamada et. al. [38]	Danish et. al. [49]	El-Souri et. al. [93]	Kallio et. al.; (2020) [19]	Varas-Doval. Et. al. [95]	Ylä-Rautio et. al. [22]	Szilvay et. al. [71]	Van Loon et. al. [52]	
Documentation mentioned but not defined	✓		✓		✓	✓	✓				✓	✓						✓		✓							
Paper-based documentation														✓					✓								
Computer based documentation		✓		✓				✓	✓	✓			✓		✓	✓	✓				✓	✓	✓	✓	✓	✓	
User completed electronic form				✓				✓							✓	✓						✓		✓	✓		
Automatically generated by computer										✓							✓										

### Facilitators and barriers

Facilitators and/or barriers that were reported mainly related to the processes of detection and classification of DRPs (n = 23) (Table 5). Although few facilitators were mentioned, the importance of this service to pharmacists and their professional identity was reported as an important facilitator. Other facilitators were an improved collaboration with the physicians, the importance of reimbursement for the service provided, an electronic support system and a high level of experience of participating pharmacists [27, 38, 52, 61, 72]. Time constraints (n = 13) and a lack of experience or trained pharmacy staff (n = 6) were the most commonly reported barriers, with fewer reporting lack of support by pharmacy owners or pharmacy staff (n = 4) and low service priority (n = 2).

**Table 5:** Facilitators and barriers for identification, classification and documentation of DRPs in community pharmacies in Europe

	Westerlund et al.; (1999) [14]	Bernsten et al.; (2001) [77]	Westerlund et al.; (2001) [28]	Layton et al.; (2002) [60]	Barris, Faus; (2003) [78]	Sturgess et al.; (2003) [16]	Chen et al.; (2005) [80]	Buurma et al.; (2006) [81]	Lynskey et al.; (2007) [83]	Krska et Avery; (2008) [55]	Montgomery et al.; (2008) [61]	Eichenberger et al.; (2010) [45]	Rossing et al.;(2012) [68]
*Facilitators*													
Re-Imbursement of service													
Importance of service to pharmacists						✔						✔	
Electronicly supported data collection													
Experience in providing pharmaceutical care services											✔		
Increase in job satisfaction through providing pharmaceutical services						✔							
Interprofessional Collaboration						✔							
*Barriers*													
Time restraint	✔	✔	✔	✔		✔	✔		✔		✔		✔
Staff shortages			✔	✔		✔							✔
Recruitments of patients		✔			✔	✔							
Lack of support of pharmacy owners					✔						✔		
Lack of experience/training			✔				✔			✔			
Low priority of the service		✔				✔							
Implementation diffculties						✔						✔	
Paper documentation													
Fear of Litigation									✔				
Logistical problems in workflows						✔							
Alert fatigue								✔					

	Ahmad et al.; (2014) [24]	Twigg et al.; (2015) [57]	Chau et al.; (2016) [72]	Heringa et al.; (2016) [47]	Messerli et al.; (2016) [63]	Maes et al.; (2017) [27]	Hamada et al.; (2019) [38]	Ylä-Rautio et al.; (2020) [22]	Szilvay et al.; (2021) [71]	Van Loon et al.; (2021) [52]
*Facilitators*										
Re-Imbursement of service			✔							
Importance of service to pharmacists										
Electronicly supported data collection						✔				
Experience in providing pharmaceutical care services										
Increase in job satisfaction through providing pharmaceutical services										
Interprofessional Collaboration										✔
*Barriers*										
Time restraint		✔				✔		✔		✔
Staff shortages										
Recruitments of patients	✔	✔			✔					
Lack of support of pharmacy owners					✔	✔				
Lack of experience/training					✔			✔	✔	
Low priority of the service										
Implementation diffculties								✔		
Paper documentation							✔			
Fear of Litigation										
Logistical problems in workflows										
Alert fatigue				✔						

## Discussion

### Statement of key findings

This scoping review identified a plethora of literature (n = 67) from across a number of European countries. These studies largely employed observational study designs. The approach to identifying DRPs varied, with professional knowledge being the most common method (n = 19), followed by identification using computer software (n = 15) and prescribing guidelines (n = 8). Forty-one studies reported the approach to classifying DRPs showcasing diverse methodologies with a lack of standardisation for the classification of DRPs in community pharmacies across Europe noted. While most studies (n = 53) reported an approach to documenting DRPs, these approaches also varied. Computer-based systems were common (n = 23), with both user-completed electronic forms and computer-generated documentation utilized. Facilitators included the importance of the service importance to pharmacists' professional identities, reimbursement, electronic support systems, and pharmacist experience. Barriers identified included time constraints, lack of staff training or experience, and inadequate support from pharmacy owners or staff.

### Strengths and weaknesses

To our knowledge this is the first review to map the broad topic of management of DRPs in European community pharmacies including the facilitators and barriers reported with them. It is strengthened by an adherence to strict PRISMA-ScR guidelines and following JBI guidance. A systematic mapping of the diverse approaches identified in the extant literature on identification, classification and documentation will facilitate the development of solutions by future researchers for their own healthcare systems.

The main limitations of this review are that firstly, few high-quality studies were identified that allowed for a direct comparison between the different approaches to identify, classify and document DRPs. Secondly, no studies were found that considered identification, classification and documentation in a single study, thereby preventing a comprehensive mapping of the entire course of DRPs in community pharmacies.

### Interpretation

There are still major differences in the focus of pharmacy curricula across Europe, with clinical pharmaceutical services often not being given as much emphasis as pharmaceutical scienes [13, 37, 38, 73]. These large differences in the training and education of pharmacists might partly explain why the lack of education and training was cited as a barrier to identify DRPs by authors. Standardized, comprehensive educational frameworks aimed at equipping pharmacists with the requisite competencies to effectively manage DRPs could address these knowledge gaps, ensuring that pharmacists are able to recognize and address potential drug-related issues in practice. Additionally, the time needed for a pharmacist to identify DRPs varies between 5 and 135 min in one study and 6 min in another study [30, 56]. Comprehensive training and a standardised procedures could help to reduce the time required and thus facilitate the implementation in practice.

The identified lack of a universally accepted DRP-classification system in community pharmacies across Europe may stem from variations in healthcare practices, regional differences, and diverse perspectives on how DRPs should be categorized [13, 30, 36]. Additionally, variations in terminology, criteria, and assessment methods used to define and categorize DRPs may also contribute [30]. Selected classification systems appear to have been used more frequently in studies from specific geographical areas, such as the Westerlund system used in Northern Europe and the PI Doc used in Germany. Although the authors did not provide any rationale for these selections, there seems to be a geographical correlation with the country of development of these instruments and the countries of use [14, 22, 61]. The complexity of the classification systems, the organisational structure within the pharmacy alongside difficulties in classifying all DRPs and using the classification system in daily practice were reported as major barriers [16, 45]. The importance of tailoring the classification system to community pharmacy practice such as PI-Doc, Westerlund and DOCUMENT has been recognised in previous research but the lack of consensus shows remaining barriers [14, 26, 74]. To address the lack of consensus across Europe, the differences in terminology and assessment methods need to be examined, involving community pharmacy practitioners in the design and development of classification systems to ensure usability in practice.

The importance of documentation is evident from the majority of studies reporting an approach to documenting DRPs. However, results also show a lack of standardised documentation and considerable heterogeneity which may be explained by the different organisational structures in healthcare settings resulting in different approaches to the documentation of DRPs. Given the differences in pharmacists' workload and healthcare structures across Europe, the time required for thorough documentation of DRPs could be a significant barrier, particularly in countries where dispensing is the main pharmaceutical service provided [17]. The use of automated computer-generated documentation could be a viable solution, as time constraints are particularly relevant for paper-based systems [14, 38]. Therefore, if adapted to the organisational conditions of community pharmacies, software solutions with high efficiency and reliability could have a positive impact on the accurate documentation of DRPs. Although few facilitators were identified in the reviewed studies, it is worth noting that a higher level of pharmacist experience facilitated the adaptation of approaches to managing DRPs and that these services were identified as important to the professional identity of the pharmacist. This is consistent with the finding that the developing clinical role of community pharmacists in countries such as the Netherlands or Switzerland was seen as a facilitator, supported by an appropriate electronic support system [38, 52, 72]. Hughes et al. (2010) noted that similar patterns of pharmaceutical care provision were identifiable in areas with similar healthcare structures and that the provision of pharmaceutical care was still emerging in different parts of Europe [75]. The recognition of the importance of pharmaceutical services in community pharmacies, as well as the changing professional identity of pharmacists, could be a driving force for meeting educational barriers.

### Further work

With the changing role of community pharmacies towards a more service-oriented role in managing DRPs, further research is warranted to tailor approaches to identifying, classifying and documenting DRPs to the different organisational structures in which community pharmacists work. In addition, this review has highlighted that lack of training and education is a barrier to managing DRPs, suggesting a potential benefit in providing a framework for future pharmacist education. As part of a dissertation project, these results are being used to examine the possibility of a uniform documentation system in the electronic health record, as an assignment for the Federal Ministry of Health. Therefore, the future focus will be on the qualitative investigation of the specific barriers and facilitators in the Austrian healthcare setting and the possibility of implementing a uniform documentation in an already existing electronic system.

## Conclusion

Despite the wealth of literature, there is a lack of a standardised approach to identifying, classifying, and documenting DRPs in European community pharmacies. The scoping review provided a valuable overview of the amount of research that has been conducted on the management of DRPs in community pharmacies. It is evident that the management of DRPs is a developing area of practice and is an expression of the evolving role profile of pharmacists around Europe. The lack of standardisation identified in this scoping review raises the question if there is indeed a need for standardisation as any standardisation of practice will need to be adaptable to the individual organisational structures of individual pharmacies and healthcare systems to avoid implementation failure. A framework for pharmacist education and a time-saving approach to documenting DRPs could overcome barriers to identifying and documenting DRP while a standardised approach to classifying DRPs could facilitate comparison of the management of DRPs across Europe.

## Supplementary Information

Below is the link to the electronic supplementary material.

Supplementary file1 (DOCX 99 KB)

Supplementary file2 (DOCX 35 KB)

Supplementary file3 (DOCX 40 KB)
