# Supplementary material for: Identification, classification, and documentation of drug related problems in community pharmacy practice in Europe: a scoping review
Source: Int J Clin Pharm. 2025 Jan 8;47(2):247–69. doi: 10.1007/s11096-024-01834-7 (PMC11920317; doi:10.1007/s11096-024-01834-7)
Supplement: Supplementary file 1 — Supplementary file1 (DOCX 99 KB) [file 11096_2024_1834_MOESM1_ESM.docx]

Identification, classification, and documentation of drug related problems in community pharmacy practice: a scoping review

# Authors

Danielle Hochhold

Innsbruck University

Department of Clinical pharmacy

+43 512 507 58882

[danielle.hochhold@uibk.ac.at](mailto:danielle.hochhold@uibk.ac.at)

Lotte Stig Nørgaard

University of Copenhagen

Department of Pharmacy

[lotte.norgaard@sund.ku.dk](mailto:lotte.norgaard@sund.ku.dk)

Derek Stewart

College of Pharmacy

QU Health

Qatar University, Doha, Qatar, PO Box 2713

(+974) 4403 5562

[d.stewart@qu.edu.qa](mailto:d.stewart@qu.edu.qa)

Anita E. Weidmann

Innsbruck University

Institute of Pharmacy

Department of Clinical Pharmacy

Tel: +43 512 507 58880

[anita.weidmann@uibk.ac.at](mailto:anita.weidmann@uibk.ac.at)

# Abstract

**Objective:**

This scoping review aims to characterise the approaches to the identification, classification, and documentation of DRPs in community pharmacies as well as any noted barriers and facilitators. It will summarize the quality measures employed in these approaches.

**Introduction:**

Drug related problems (DRPs) can cause damage to patient´s health, increase hospital admissions and healthcare costs. The identification, classification and documentation of these problems can improve patient care and inter-professional communication. Community pharmacies are well placed to identify DRPs. There are validated methods to document and classify drug-related problems such as the Pharmaceutical Care Network Europe Classification system or the Westerlund system, but to our knowledge there is no standardized way of documenting and classifying DRPs in community pharmacies across Europe and different countries use different approaches.

**Inclusion criteria:**

All study designs, opinion papers, case reports, editorials, dissertations and published conference abstracts about the identification, classification, and documentation of DRPs in community pharmacies across Europe since inception will be included.

**Methods:** Pubmed, Web of Science, Cochrane library, Embase, Social Science Research Network (SSRN), Cumulative Index to Nursing and Allied Health Literature (CINAHL), Psycinfo, Open Dissertation, Livivo, openAire., will be included. In addition, International Pharmaceutical Abstracts (IPA) and published conference abstracts will be included.

Article selection will be carried out by two researchers independently (DH, AEW), disagreement will be resolved by discussion within the research team (LN, DS). Three highly experienced research academics, all of whom have extensive experience in conducting scoping and systematic literature reviews. Data extraction will be performed by one researcher and independently checked by a second researcher. Data will be charted and reported using a tool developed for the purpose of this review. Reporting of this scoping review will follow the Preferred Reporting Items for Systematic Reviews extension for scoping reviews (PRISMA-ScR).

# Introduction

According to Pharmaceutical Care Network Europe (PCNE) Classification V 9.1, a drug-related problem (DRP) is "an event or circumstance related to drug therapy that actually or potentially affects desired health outcomes."^1^ This definition includes unnecessary or inappropriate drug treatments, adverse drug reactions, dosages, or poor adherence. Such drug-related problems are often caused by medication errors (either a prescription error or an error in the use or administration), inappropriate over-the-counter medications (e.g., incorrect dosage, incorrect drug, incorrect indication) or poor health literacy/compliance^1^. DRPs can cause damage to patient’s health, increase hospital admissions, and raise healthcare costs^2,3^.

While the roles of pharmacist vary across settings and countries there has been a general shift in the community pharmacist’s role from a product-supply function to a more patient centred role^4,5^. Nowadays one of their professional commitments in many countries is to identify DRPs and make recommendations to patients, caregivers, and doctors^6-13^. Community pharmacists are well-placed to detect and prevent or resolve DRPs because they are easily accessible to the public and in many countries have a close cooperation with general practitioners and other healthcare providers^14^. Different studies have highlighted that the identification and management of DRPs is commonplace in some but not all countries^14-18^. Westerlund et al. (2009) stated that the management of DRPs increases the attention of healthcare providers towards patients-needs and leads to increased patient safety and decreased healthcare costs^19^.

The classification and documentation of DRPs by pharmacists has been shown to improve effectiveness and efficiency of pharmaceutical care as well as inter-professional communication ^19-21^. As noted by Van Mil et al in 2004 the documentation instrument should be well-constructed and validated^22^. The validation of the documentation and classification system is important to ensure that it collects the desired information accurately and completely and that the documentation instrument is easily understood by pharmacists and researchers to avoid introduction of bias^22,23^. The need for a valid method to document and classify pharmaceutical interventions has been recognised for more than 20 years by different researchers and validated methods to document and classify DRPs have been developed^24,25^. These include the Westerlund system or the Pharmaceutical Care Network Europe (PCNE) Classification system as well as the PI-Doc system in Germany or the ClinPhADoc system in Switzerland ^1,16,20,24,26^. Nonetheless there is still no standardized way of documentation and classification of DRPs in community pharmacy practice with different countries using different documentation methods^8,16,17^.

Although many studies show that community pharmacists recognize and respond to DRPs and the need for classification and documentation of DRPs is established, there is little published research on how community pharmacies identify, classify and document DRPs in practice and what the facilitators or barriers in doing so are^5,16,17,20,27-34^. This scoping review aims to detail the different methods to identify, classify and document DRPs in community pharmacies as well as any noted barriers and facilitators. It will summarize and critically appraise the Quality and Validity of the different models used.

# Review questions

This scoping review aims to characterise the approaches to the identification, classification and documentation of DRPs in community pharmacies across Europe.

Specific questions

- What are the approaches?
- What are the reported facilitators and barriers to these approaches?
- What quality measures are employed in these approaches?

# Keywords

Community pharmacy, documentation, drug related problems, pharmacy services

# Eligibility criteria

### Participants

Employees in community pharmacies across Europe

Inclusion / Exclusion Criteria

Inclusion criteria: any articles reporting aspects of the identification, classification, and documentation of drug related problems (prescription medication as well as pharmacy only and over the counter medication) in community pharmacies across Europe. Drug selection (duplicate drug, drug interaction, wrong drug, wrong dosage, wrong strength, contraindication, no indication); compliance (erratic use of medication, drug misuse, other compliance problems); untreated conditions; adverse drug reactions and the prevention of these; inappropriate use of OTC medication.

Exclusion criteria: there are no exclusion criteria.

### Concept

As stated in the JBI Manual for Evidence Synthesis a scoping review is undertaken to map the current evidence in a specific field and to identify knowledge gaps^35^.The concept of this scoping review is drug related problems in community pharmacies.

### Context

As this is the first phase of a piece of research aimed at determining the number and types of DRPs noted in community pharmacy practice across Austria, this review focuses on the reporting of these methods across Europe. Studies published in the English language from inception will be included.

### Types of Sources

This scoping review will consider all study designs.

Text and opinion papers will also be considered for inclusion in this scoping review. Grey literature such as case reports, editorials, discussion papers, published conference proceedings, dissertations and theses will also be included.

# Methods

The proposed scoping review will be conducted in accordance with the JBI methodology for scoping reviews and carried out according to the PRISMA ScR reporting guidelines^35,36^.

### Search strategy

A research librarian will be consulted to optimise the keyword search and keyword strings.

As a first step Pubmed will be searched with the following keywords and index terms: “community pharmac*” AND “drug-related-problem*” AND (“documentation” OR “identification” OR “classification”)

The second search will be using the keywords above and all additional keywords/index terms found in the first search. This search will be carried out at: Pubmed, Web of Science, Cochrane library, Embase, Social Science Research Network (SSRN), Cumulative Index to Nursing and Allied Health Literature (CINAHL), Psycinfo, Open Dissertation, Livivo, openAire., will be included. In addition, International Pharmaceutical Abstracts (IPA) and published conference abstracts will be included.

The reference lists of included full text and other key studies will be hand-searched for further eligible studies for inclusion and citation searching will also be carried out using the included full texts to identify further relevant research.

### Study/Source of Evidence selection

- Two researchers (DH, AW, DS, LN) will independently screen the titles, abstract and full text for inclusions and exclusion criteria. Disagreements will be resolved by discussion. If agreement cannot be reached, a third researcher will be included in the phase.
- Endnote web and Excel will be used.

### Data Extraction

- The identification, screening, eligibility, and inclusion of papers will be performed and presented according to the JBI Manual for evidence synthesis^35^ and the PRISMA flow diagram^36^ (2020). The extraction tools will be designed according to the objectives in this scoping review.
- Data extraction will be performed by one researcher (DH) and checked by another researcher (AW, DS, LN) independently. All disagreements will be solved by discussion within the research team.
- Extracted data will include: title, date of publication, country, population (how many pharmacists), timeframe of documentation, approaches to identify, classify and document DRPs, reported facilitators and barriers, quality measures of these approaches
  - What are the approaches? Extracted data: what approaches are used for identifying, documenting and classifying DRPs, paper-based or electronical
  - What are the reported facilitators and barriers to these approaches? Extracted data: reported facilitators and barriers, how were they reported
  - What quality measures are employed in these approaches? Extracted data: validation process, piloting phase
- Authors will not be contacted for unreported data and any missing data will be reported as missing in the data extraction form.
- Excel and Word will be used for data extraction.

### Data Analysis and Presentation

This scoping review will detail the different methods to identify, classify and document DRPs in community pharmacies as well as any noted barriers and facilitators.

The charted evidence will be presented in a draft chart according to the PCC inclusion criteria. The chart will be accompanied by a descriptive summary of the findings and any gaps in knowledge that appear.

# Acknowledgements

# Funding

No funding

# Conflicts of interest

No conflict of interest

# References

1. van Mil JWF, Horvat N, Westerlund T, et al. The PCNE Classification V 9.1. Pharmaceutical Care Network Europe Association: 2020.

2. Ni XF, Yang CS, Bai YM, et al. Drug-Related Problems of Patients in Primary Health Care Institutions: A Systematic Review. Front Pharmacol 2021;12(698907

3. Royal S, Smeaton L, Avery AJ, et al. Interventions in primary care to reduce medication related adverse events and hospital admissions: systematic review and meta-analysis. Qual Saf Health Care 2006;15(1):23-31

4. Yong FR, Garcia-Cardenas V, Williams KA, et al. Factors affecting community pharmacist work: A scoping review and thematic synthesis using role theory. 2020;16(2):123-141

5. Al-Taani GM, Ayoub NM. Community pharmacists' routine provision of drug-related problem-reduction services. PLoS One 2022;17(5):e0267379, doi:10.1371/journal.pone.0267379

6. Ang F, Pau JE, Koh EW, et al. Drug-related problems associated with community-dwelling older persons living alone in Singapore. Int J Clin Pharm 2019;41(3):719-727, doi:10.1007/s11096-019-00813-7

7. Ax F, Brånstad JO, Westerlund T. Pharmacy counselling models: a means to improve drug use. J Clin Pharm Ther 2010;35(4):439-51, doi:10.1111/j.1365-2710.2009.01138.x

8. Basger BJ, Moles RJ, Chen TF. Application of drug-related problem (DRP) classification systems: a review of the literature. Eur J Clin Pharmacol 2014;70(7):799-815, doi:10.1007/s00228-014-1686-x

9. Bedouch P, Charpiat B, Conort O, et al. Assessment of clinical pharmacists' interventions in French hospitals: results of a multicenter study. Ann Pharmacother 2008;42(7):1095-103, doi:10.1345/aph.1L045

10. Bekele F, Tsegaye T, Negash E, et al. Magnitude and determinants of drug-related problems among patients admitted to medical wards of southwestern Ethiopian hospitals: A multicenter prospective observational study. PLoS One 2021;16(3):e0248575, doi:10.1371/journal.pone.0248575

11. Casper EA, El Wakeel LM, Saleh MA, et al. Management of pharmacotherapy-related problems in acute coronary syndrome: Role of clinical pharmacist in cardiac rehabilitation unit. Basic Clin Pharmacol Toxicol 2019;125(1):44-53, doi:10.1111/bcpt.13210

12. Castronovo A, Gervais F, Mongaret C, et al. Pharmacists' interventions on prescription problems in one French community pharmacy: A prospective pilot study. Ann Pharm Fr 2018;76(4):299-305, doi:10.1016/j.pharma.2018.02.002

13. Chan DC, Chen JH, Wen CJ, et al. Effectiveness of the medication safety review clinics for older adults prescribed multiple medications. J Formos Med Assoc 2014;113(2):106-13, doi:10.1016/j.jfma.2012.04.013

14. van Loon WEE, Borgsteede SDS, Baas GWG, et al. Nature and frequency of prescription modifications in community pharmacies: A nationwide study in the Netherlands. Br J Clin Pharmacol 2021;87(3):1455-1465, doi:10.1111/bcp.14548

15. Ylä-Rautio H, Siissalo S, Leikola S. Drug-related problems and pharmacy interventions in non-prescription medication, with a focus on high-risk over-the-counter medications. Int J Clin Pharm 2020;42(2):786-795

16. Hamada N, Quintana Bárcena P, Maes KA, et al. Clinical Pharmacy Activities Documented (ClinPhADoc): Development, Reliability and Acceptability of a Documentation Tool for Community Pharmacists. Pharmacy (Basel) 2019;7(4), doi:10.3390/pharmacy7040162

17. Maes KA, Bruch S, Hersberger KE, et al. Documentation of pharmaceutical care: development of an intervention oriented classification system. Int J Clin Pharm 2017;39(2):354-363, doi:10.1007/s11096-017-0442-6

18. Vik S, Weidemann P, Gangas IEM, et al. Pharmaceutical interventions on prescriptions in Norwegian community and hospital pharmacies. Int J Clin Pharm 2021;43(4):872-877, doi:10.1007/s11096-020-01188-w

19. Westerlund T, Gelin U, Pettersson E, et al. A retrospective analysis of drug-related problems documented in a national database. Int J Clin Pharm 2013;35(2):202-9, doi:10.1007/s11096-012-9724-1

20. Westerlund T, Marklund B. Assessment of the clinical and economic outcomes of pharmacy interventions in drug-related problems. J Clin Pharm Ther 2009;34(3):319-27, doi:10.1111/j.1365-2710.2008.01017.x

21. Westerlund T, Brånstad JO. GPs' views on patient drug use and the pharmacist's role in DRP management. Pharm World Sci 2010;32(5):562-5, doi:10.1007/s11096-010-9408-7

22. van Mil JW, Westerlund LO, Hersberger KE, et al. Drug-related problem classification systems. Ann Pharmacother 2004;38(5):859-67, doi:10.1345/aph.1D182

23. Maes KA, Tremp RM, pharmacy GWgoc, et al. Demonstrating the clinical pharmacist's activity: validation of an intervention oriented classification system. Int J Clin Pharm 2015;37(6):1162-71, doi:10.1007/s11096-015-0179-z

24. Lacy CF, Saya FG, Shane RR. Quality of pharmacists' documentations in patients' medical records. Am J Health Syst Pharm 1996;53(18):2171-5, doi:10.1093/ajhp/53.18.2171

25. Westerlund T, Allebeck P, Marklund B, et al. Evaluation of a model for counseling patients with dyspepsia in Swedish community pharmacies. Am J Health Syst Pharm 2003;60(13):1336-41

26. Schaefer M. Discussing basic principles for a coding system of drug-related problems: the case of PI-Doc. Pharm World Sci 2002;24(4):120-7, doi:10.1023/a:1019543029936

27. Al-Hajje AH, Atoui F, Awada S, et al. Drug-related problems identified by clinical pharmacist's students and pharmacist's interventions. Ann Pharm Fr 2012;70(3):169-76, doi:10.1016/j.pharma.2012.02.004

28. Avery AJ, Rodgers S, Cantrill JA, et al. A pharmacist-led information technology intervention for medication errors (PINCER): a multicentre, cluster randomised, controlled trial and cost-effectiveness analysis. Lancet 2012;379(9823):1310-9

29. Basheti IA, Tadros OK, Aburuz S. Value of a Community-Based Medication Management Review Service in Jordan: A Prospective Randomized Controlled Study. Pharmacotherapy 2016;36(10):1075-1086, doi:10.1002/phar.1833

30. DeName B, Divine H, Nicholas A, et al. Identification of medication-related problems and health care provider acceptance of pharmacist recommendations in the DiabetesCARE program. Journal of the American Pharmacists Association : JAPhA 2008;48(6):731-736, doi:10.1331/JAPhA.2008.07070

31. Gernant SA, Zillich AJ, Snyder ME. Access to Medical Records' Impact on Community Pharmacist-Delivered Medication Therapy Management: A Pilot From the Medication Safety Research Network of Indiana (Rx-SafeNet). Journal of pharmacy practice 2018;31(6):642-650, doi:10.1177/0897190017735422

32. Hämmerlein A, Griese N, Schulz M. Survey of drug-related problems identified by community pharmacies. Ann Pharmacother 2007;41(11):1825-32, doi:10.1345/aph.1K207

33. Maes KA, Ruppanner JA, Imfeld-Isenegger TL, et al. Dispensing of Prescribed Medicines in Swiss Community Pharmacies-Observed Counselling Activities. Pharmacy (Basel, Switzerland) 2018;7(1), doi:10.3390/pharmacy7010001

34. Williams M, Peterson GM, Tenni PC, et al. DOCUMENT: a system for classifying drug-related problems in community pharmacy. Int J Clin Pharm 2012;34(1):43-52, doi:10.1007/s11096-011-9583-1

35. Aromataris E, Munn Z. JBI Manual for Evidence Synthesis. 2020.

36. Page MJ, McKenzie JE, Bossuyt PM, et al. The PRISMA 2020 statement: an updated guideline for reporting systematic reviews. BMJ 2021;372(n71, doi:10.1136/bmj.n71

37. Centre TENaUE. Enhancing the QUAlity and Transparency Of health Research. 2023. Available from: <https://www.equator-network.org> [Last Accessed; 16.01.2023].
