# Supplementary material for: Identification, classification, and documentation of drug related problems in community pharmacy practice in Europe: a scoping review
Source: Int J Clin Pharm. 2025 Jan 8;47(2):247–69. doi: 10.1007/s11096-024-01834-7 (PMC11920317; doi:10.1007/s11096-024-01834-7)
Supplement: Supplementary file 2 — Supplementary file2 (DOCX 35 KB) [file 11096_2024_1834_MOESM2_ESM.docx]

Scoping review search strategy as applied to each database

| **database** | query | field | hits |  |
| --- | --- | --- | --- | --- |
| **Pubmed** |  |  |  |  |
|  | "drug related problem*" | All | 1937 |  |
|  | "drug therapy problem*" | All | 244 |  |
|  | "medication related problem*" | All | 655 |  |
|  | "medicine related problem*" | All | 43 |  |
|  | "therapy related problem*" | All | 25 |  |
|  | "medication management problem*" | All | 18 |  |
|  | "intervention*" | All | 1423984 |  |
|  | "drug related issue*" | All | 77 |  |
|  | "drug related finding*" | All | 9 |  |
|  | "medication review" | All | 2336 |  |
|  | "drug utilisation review" | All | 26 |  |
|  | "drug utilization review" | All | 4164 |  |
|  | "pharmaceutical care" | All | 5741 |  |
|  | "pharmaceutical service*" | All | 16176 |  |
|  | "domiciliary medication review" | All | 7 |  |
|  | 1 OR 2 OR 3 OR 4 OR 5 OR 6 OR7 OR 8 OR 9 OR 10 OR 11 OR 12 OR 13 OR 14 OR 15 |  | 147881 |  |
|  | "retail pharmac*" | All | 812 |  |
|  | "community pharmac*" | All | 11513 |  |
|  | "community pharmacy services" [mesh] | All | 5669 |  |
|  | 16 OR 17 OR 18 |  | 12171 |  |
|  | 20 AND 26 |  | 4448 |  |
|  | english |  | 4269 |  |
| **Web of science** |  |  |  |  |
|  | "drug related problem*" | All | 2557 |  |
|  | "drug therapy problem*" | All | 244 |  |
|  | "medication related problem*" | All | 668 |  |
|  | "medicine related problem*" | All | 52 |  |
|  | "therapy related problem*" | All | 27 |  |
|  | "medication management problem*" | All | 13 |  |
|  | "intervention*" | All | 1633071 |  |
|  | "drug related issue*" | All | 91 |  |
|  | "drug related finding*" | All | 6 |  |
|  | "medication review" | All | 1963 |  |
|  | "drug utilisation review" | All | 44 |  |
|  | "drug utilization review" | All | 435 |  |
|  | "pharmaceutical care" | All | 5139 |  |
|  | "pharmaceutical service*" | All | 2117 |  |
|  | "domiciliary medication review" | All | 5 |  |
|  | 1 OR 2 OR 3 OR 4 OR 5 OR 6 OR7 OR 8 OR 9 OR 10 OR 11 OR 12 OR 13 OR 14 OR 15 |  | 1640876 |  |
|  | "retail pharmac*" | All | 887 |  |
|  | "community pharmac*" | All | 11551 |  |
|  | "community pharmacy services" | All | 12248 |  |
|  |  |  | 19764 |  |
|  | 20 AND 26 |  | 6441 |  |
|  | english |  | 6053 |  |
| **Cochrane library** |  |  |  |  |
|  | "drug related problem*" | phrase | 440 |  |
|  | "drug therapy problem*" | phrase | 52 |  |
|  | "medication related problem*" | phrase | 116 |  |
|  | "medicine related problem*" | phrase | 13 |  |
|  | "therapy related problem*" | phrase | 7 |  |
|  | "medication management problem*" | phrase | 6 |  |
|  | "intervention*" | phrase | 566844 |  |
|  | "drug related issue*" | phrase | 12 |  |
|  | "drug related finding*" | phrase | 1 |  |
|  | "medication review" | phrase | 756 |  |
|  | "drug utilisation review" | phrase | 2 |  |
|  | "drug utilization review" | phrase | 258 |  |
|  | "pharmaceutical care" | phrase | 967 |  |
|  | "pharmaceutical service*" | phrase | 385 |  |
|  | "domiciliary medication review" | phrase | 3 |  |
|  | 1 OR 2 OR 3 OR 4 OR 5 OR 6 OR7 OR 8 OR 9 OR 10 OR 11 OR 12 OR 13 OR 14 OR 15 |  | 567467 |  |
|  | "retail pharmac*" | phrase | 66 |  |
|  | "community pharmac*" | phrase | 1495 |  |
|  | "community pharmacy servic*" | phrase | 412 |  |
|  | 16 OR 17 OR 18 |  | 1553 |  |
|  | 20 AND 26 |  | 1222 |  |
|  | english |  | 1196 |  |
| **SSRN** |  |  |  |  |
|  | pharmac* | Title, Abstract keywords, fulltext | 106 |  |
|  | drug | within 1 | 22 |  |
|  | medication | within 1 | 1 |  |
|  | medicine | within 1 | 10 |  |
|  | therapy | within 1 | 4 |  |
|  | intervention | within 1 | 4 |  |
|  | care | within 1 | 2 |  |
| **Psycinfo** |  |  |  | |
|  | "drug related problem*" | All |  | |
|  | "drug therapy problem*" | All |  | |
|  | "medication related problem*" | All |  | |
|  | "medicine related problem*" | All |  | |
|  | "therapy related problem*" | All |  | |
|  | "medication management problem*" | All |  | |
|  | "intervention*" | All |  | |
|  | "drug related issue*" | All |  | |
|  | "drug related finding*" | All |  | |
|  | "medication review" | All |  | |
|  | "drug utilisation review" | All |  | |
|  | "drug utilization review" | All |  | |
|  | "pharmaceutical care" | All |  | |
|  | "pharmaceutical service*" | All |  | |
|  | "domiciliary medication review" | All |  | |
|  | 1 OR 2 OR 3 OR 4 OR 5 OR 6 OR7 OR 8 OR 9 OR 10 OR 11 OR 12 OR 13 OR 14 OR 15 |  | 520434 | |
|  | "retail pharmac*" | All |  | |
|  | "community pharmac*" | All |  | |
|  | DE"pharmacy" | All |  | |
|  | 17 OR 18 OR 19 |  | 2350 | |
|  |  |  |  | |
|  | 16 AND 20 |  | 895 | |
|  | english |  | 791 | |
| **Open dissertation** |  |  |  | |
|  | "drug related problem*" | All |  | |
|  | "drug therapy problem*" | All |  | |
|  | "medication related problem*" | All |  | |
|  | "medicine related problem*" | All |  | |
|  | "therapy related problem*" | All |  | |
|  | "medication management problem*" | All |  | |
|  | "intervention*" | All |  | |
|  | "drug related issue*" | All |  | |
|  | "drug related finding*" | All |  | |
|  | "medication review" | All |  | |
|  | "drug utilisation review" | All |  | |
|  | "drug utilization review" | All |  | |
|  | "pharmaceutical care" | All |  | |
|  | "pharmaceutical service*" | All |  | |
|  | "domiciliary medication review" | All |  | |
|  | 1 OR 2 OR 3 OR 4 OR 5 OR 6 OR7 OR 8 OR 9 OR 10 OR 11 OR 12 OR 13 OR 14 OR 15 |  | 50066 | |
|  | "retail pharmac*" | All |  | |
|  | "community pharmac*" | All |  | |
|  | "pharmacy services" [mesh] | All |  | |
|  | 17 OR 18 OR 19 |  | 465 | |
|  |  |  |  | |
|  | 16 AND 20 |  | 131 | |
|  | english |  | 131 | |
| **Livivo** |  |  |  | |
|  | (drug related problem) | open search |  | |
|  | (drug therapy problem) | open search |  | |
|  | (medication related problem) | open search |  | |
|  | (medicine related problem) | open search |  | |
|  | (therapy related problem) | open search |  | |
|  | (medication management problem) | open search |  | |
|  | (intervention) | open search |  | |
|  | (drug related issue) | open search |  | |
|  | (drug related finding) | open search |  | |
|  | (medication review) | open search |  | |
|  | (drug utilisation review) | open search |  | |
|  | (drug utilization review) | open search |  | |
|  | (pharmaceutical care) | open search |  | |
|  | (pharmaceutical service) | open search |  | |
|  | (domiciliary medication review) | open search |  | |
|  | 1 OR 2 OR 3 OR 4 OR 5 OR 6 OR7 OR 8 OR 9 OR 10 OR 11 OR 12 OR 13 OR 14 OR 15 |  | 2271038 | |
|  | (retail pharmacy) OR (retail pharmacies) | open search |  | |
|  | (community pharmacy) OR (community pharmacies) | open search |  | |
|  |  |  |  | |
|  | 17 OR 18 |  | 21599 | |
|  |  |  |  | |
|  | 16 AND 19 |  | 21506 | |
|  | english |  | 19904 | |
| **openAire** |  |  |  | |
|  | "drug related problem*" | Any field |  | |
|  | "drug therapy problem*" | Any field |  | |
|  | "medication related problem*" | Any field |  | |
|  | "medicine related problem*" | Any field |  | |
|  | "therapy related problem*" | Any field |  | |
|  | "medication management problem*" | Any field |  | |
|  | "intervention*" | Any field |  | |
|  | "drug related issue*" | Any field |  | |
|  | "drug related finding*" | Any field |  | |
|  | "medication review" | Any field |  | |
|  | "drug utilisation review" | Any field |  | |
|  | "drug utilization review" | Any field |  | |
|  | "pharmaceutical care" | Any field |  | |
|  | "pharmaceutical service*" | Any field |  | |
|  | "domiciliary medication review" | Any field |  | |
|  | 1 OR 2 OR 3 OR 4 OR 5 OR 6 OR7 OR 8 OR 9 OR 10 OR 11 OR 12 OR 13 OR 14 OR 15 |  | 1842251 | |
|  | community AND pharmacy | Any field |  | |
|  | retail AND pharmacy | Any field |  | |
|  | 17 OR 18 OR 19 |  |  | |
|  |  |  |  | |
|  | 16 AND 20 |  | 7573 | |
|  | english |  | 1840 | |
| **ProQuest** |  |  |  | |
|  | "drug related problem*" | Any field |  | |
|  | "drug therapy problem*" | Any field |  | |
|  | "medication related problem*" | Any field |  | |
|  | "medicine related problem*" | Any field |  | |
|  | "therapy related problem*" | Any field |  | |
|  | "medication management problem*" | Any field |  | |
|  | "intervention*" | Any field |  | |
|  | "drug related issue*" | Any field |  | |
|  | "drug related finding*" | Any field |  | |
|  | "medication review" | Any field |  | |
|  | "drug utilisation review" | Any field |  | |
|  | "drug utilization review" | Any field |  | |
|  | "pharmaceutical care" | Any field |  | |
|  | "pharmaceutical service*" | Any field |  | |
|  | "domiciliary medication review" | Any field |  | |
|  | 1 OR 2 OR 3 OR 4 OR 5 OR 6 OR7 OR 8 OR 9 OR 10 OR 11 OR 12 OR 13 OR 14 OR 15 |  | 997427 | |
|  |  |  |  | |
|  |  |  |  | |
|  | "retail pharmac*" | Any field |  | |
|  | "community pharmac*" | Any field |  | |
|  |  |  |  | |
|  | 17 OR 18 |  | 9010 | |
|  |  |  |  | |
|  | 16 AND 20 |  | 6606 | |
|  | english+europe |  | 1037 | |
| **CINAHL** |  |  |  | |
|  | "drug related problem*" | All text | 2607 | |
|  | "drug therapy problem*" | All text | 462 | |
|  | "medication related problem*" | All text | 1238 | |
|  | "medicine related problem*" | All text | 118 | |
|  | "therapy related problem*" | All text | 44 | |
|  | "medication management problem*" | All text | 55 | |
|  | "intervention*" | All text | 997309 | |
|  | "drug related issue*" | All text | 204 | |
|  | "drug related finding*" | All text | 0 | |
|  | "medication review" | All text | 4754 | |
|  | "drug utilisation review" | All text | 35 | |
|  | "drug utilization review" | All text | 378 | |
|  | "pharmaceutical care" | All text | 5497 | |
|  | "pharmaceutical service*" | All text | 5610 | |
|  | "domiciliary medication review" | All text | 21 | |
|  | 1 OR 2 OR 3 OR 4 OR 5 OR 6 OR7 OR 8 OR 9 OR 10 OR 11 OR 12 OR 13 OR 14 OR 15 |  | 1007399 | |
|  | "retail pharmac*" |  | 426 | |
|  | "community pharmac*" |  | 15201 | |
|  |  |  |  | |
|  | 17 OR 18 |  | 15573 | |
|  | 16 AND 19 | apply related words, apply equivalent subjects | 6819 | |
|  | english |  | 6799 | |
|  | without medline |  | 4771 | |
|  | Continental Europe, Europe, Middle East, UK & Ireland |  | 1334 | |
| **IPA** |  |  |  | |
|  |  |  |  | |
|  | "drug related problem*" | All text |  | |
|  | "drug therapy problem*" | All text |  | |
|  | "medication related problem*" | All text |  | |
|  | "medicine related problem*" | All text |  | |
|  | "therapy related problem*" | All text |  | |
|  | "medication management problem*" | All text |  | |
|  | "intervention*" | All text |  | |
|  | "drug related issue*" | All text |  | |
|  | "drug related finding*" | All text |  | |
|  | "medication review" | All text |  | |
|  | "drug utilisation review" | All text |  | |
|  | "drug utilization review" | All text |  | |
|  | "pharmaceutical care" | All text |  | |
|  | "pharmaceutical service*" | All text |  | |
|  | "domiciliary medication review" | All text |  | |
|  | 1 OR 2 OR 3 OR 4 OR 5 OR 6 OR7 OR 8 OR 9 OR 10 OR 11 OR 12 OR 13 OR 14 OR 15 |  | 61530 | |
|  | "retail pharmac*" |  |  | |
|  | "community pharmac*" |  |  | |
|  |  |  |  | |
|  | 17 OR 18 |  | 13017 | |
|  | 16 AND 19 | apply related words | 3779 | |
