# Supplementary material for: Identification, classification, and documentation of drug related problems in community pharmacy practice in Europe: a scoping review
Source: Int J Clin Pharm. 2025 Jan 8;47(2):247–69. doi: 10.1007/s11096-024-01834-7 (PMC11920317; doi:10.1007/s11096-024-01834-7)
Supplement: Supplementary file 3 — Supplementary file3 (DOCX 40 KB) [file 11096_2024_1834_MOESM3_ESM.docx]

**SCOPING REVIEW – Data extraction supplementary material 3**

| Datenerhebung ScR |  |  |  |  |
| --- | --- | --- | --- | --- |
| Title |  |  |  |  |
| Author |  |  |  |  |
| Journal |  |  |  |  |
| Year of publication |  |  |  |  |
| Country |  |  |  |  |
| Aim |  |  |  |  |
| Design |  |  |  |  |
| Method |  |  |  |  |
| Study population |  |  |  |  |
| Number of participants (response rate) |  |  |  |  |
| Approach to identification of DRPs |  |  |  |  |
| Approach to classification of DRPs |  |  |  |  |
| Approach to documentation of DRPs |  |  |  |  |
| Definitions of DRP |  |  |  |  |
| Follow up of patients after suggested DRP intervention |  |  |  |  |
| Facilitators of service |  |  |  |  |
| Barriers of service |  |  |  |  |
| Measures taken to quality assure method |  |  |  |  |
| Outcome reported |  |  |  |  |
| Key findings |  |  |  |  |
| Conclusion |  |  |  |  |
| Comments |  |  |  |  |
